# Dosimetric and robustness evaluation of biaxially rotational dynamic radiation therapy with robust optimization

**DOI:** 10.1002/acm2.70194

**Published:** 2025-08-08

**Authors:** Kouta Hirotaki, Kana Motegi, Shunsuke Moriya, Masashi Wakabayashi, Takeji Sakae, Masashi Ito, Sadamoto Zenda

**Affiliations:** ^1^ Doctoral Program in Medical Sciences, Graduate School of Comprehensive Human Sciences University of Tsukuba Ibaraki Japan; ^2^ Department of Radiological Technology National Cancer Center Hospital East Chiba Japan; ^3^ Section of Radiation Safety and Quality Assurance National Cancer Center Hospital East Chiba Japan; ^4^ Faculty of Medicine University of Tsukuba Ibaraki Japan; ^5^ Biostatistics Division Center for Research Administration and Support National Cancer Center Hospital East Kashiwa Japan; ^6^ Department of Radiation Oncology National Cancer Center Hospital East Kashiwa Japan

**Keywords:** biaxially rotational dynamic radiation therapy (BROAD-RT), dynamic trajectory, Head and neck cancer, Non‐coplanar, robust optimization, robust planning, VMAT

## Abstract

**Background:**

Studies suggest that integrating robust planning and noncoplanar volumetric modulated arc therapy (VMAT) may be a useful strategy for maximizing the benefits of dose delivery while maintaining resilience to uncertainty; however, the robustness of target coverage and the dose‐sparing performance for organs at risk of techniques that combine dynamic trajectory irradiation and robust planning have not been fully investigated.

**Purpose:**

We aimed to evaluate the combination of biaxially rotational dynamic radiation therapy (BROAD‐RT) with robust planning to improve the dosimetric outcomes and robustness of head and neck cancer (HNC) radiation therapy.

**Methods:**

We retrospectively analyzed 10 patients with oropharyngeal and hypopharyngeal cancers who were treated with VMAT. Robust‐VMAT, Robust‐Non‐Coplanar‐VMAT (Robust‐NC‐VMAT), and Robust‐BROAD‐RT plans were generated using the RayStation treatment planning system, with patient setup uncertainties of 3–5 mm in each of the three axial directions (left–right, anterior–posterior, and superior–inferior) applied to the clinical target volume (CTV) during robust optimization. The prescribed doses were 70 and 56 Gy in 35 fractions for high‐ and low‐risk CTVs, respectively. Dosimetric parameters, including dose coverage for the CTVs and organ at risk (OAR) sparing, were compared between the plans. Robustness was evaluated using eight uncertainty scenarios, each generated by applying ± 1.7 mm translational shifts simultaneously along all three orthogonal axes (left–right, anterior–posterior, and superior–inferior), resulting in a combined vector displacement of approximately 3 mm.

**Results:**

The Robust‐BROAD‐RT plans significantly reduced the mean doses to the contralateral and ipsilateral parotid glands and the oral cavity by 8.17, 6.20, and 3.03 Gy, respectively, compared to the Robust‐VMAT plans (*p* < 0.001, *p* < 0.001, and *p* = 0.002, respectively). Similarly, the Robust‐BROAD‐RT plans achieved significantly lower doses to the parotid glands and the oral cavity compared to the Robust‐NC‐VMAT plans (*p* = 0.004, *p* = 0.014, and *p* = 0.014, respectively). No significant differences were observed in the target coverage indices (D98, D50), conformity index (CI), or homogeneity index (HI) among the three techniques. The worst‐case scenario analysis showed that the degree of deterioration in these target‐related parameters did not differ significantly among the Robust‐BROAD‐RT, Robust‐VMAT, and Robust‐NC‐VMAT plans.

**Conclusions:**

The Robust‐BROAD‐RT plan achieved a further reduction in doses to the parotid and oral cavities while maintaining robustness comparable to that of conventional Robust VMAT planning.

## 1 INTRODUCTION

Head and neck cancers (HNCs) are one of the most complex malignancies to treat because of their proximity to critical organs such as the spinal cord, salivary glands, and oral cavity. However, the development of intensity‐modulated radiation therapy (IMRT) has revolutionized HNC treatment, enabling precise dose delivery to tumors while sparing the surrounding normal tissues. Clinical trials and retrospective studies have demonstrated superior locoregional control and reduced toxicities with IMRT compared with conventional techniques.^1^, ^2^ Despite its efficacy, implementation of IMRT remains challenging. Particularly, the intricate anatomy and dynamic changes in patient geometry during treatment, including tumor shrinkage, weight loss, and variations in organ positioning, can lead to suboptimal dose distributions over the treatment course.^3^, ^4^ Although six‐degree‐of‐freedom (6DoF) couch systems are commonly used to correct patient positioning errors during IMRT, residual errors persist in the head and neck region owing to nonrigid deformations of the cervical spine curvature, which adversely affect dose distribution.^5^

Therefore, robust planning that incorporates uncertainty margins directly into the optimization process to create dose distributions that are resilient to geometric and positional variations has been implemented.^6^ This technique has been successfully employed at various anatomical sites, including the head and neck, to mitigate the effects of inter‐ and intrafractional variations.^7^, ^8^ However, robust planning imposes stricter constraints during treatment planning, often making it challenging to achieve optimal trade‐offs between key dosimetric parameters and organ at risk (OAR) sparing. Studies have also reported instances wherein robust optimization compromises critical dosimetric endpoints such as the homogeneity index (HI) and conformity index (CI), particularly in anatomically complex regions such as the head and neck.^9^, ^10^ In this regard, volumetric modulated arc therapy (VMAT), particularly non‐coplanar VMAT, has further improved dose distribution by introducing greater flexibility in beam delivery. Non‐coplanar trajectories enhance the CI and HI, enabling superior tumor coverage and better sparing of OARs compared with conventional coplanar approaches.^11^, ^12^ Early clinical experience suggests that non‐coplanar VMAT may be especially beneficial in HNCs, where OAR proximity poses significant planning challenges.^13^, ^14^ Thus, the integration of robust planning and non‐coplanar VMAT may be a useful strategy for maximizing the benefits of dose delivery while maintaining resilience to uncertainty. However, further studies are required to validate its clinical utility and feasibility.

The OXRAY system (Hitachi High‐Tech, Tokyo, Japan) is a significant advancement in radiation therapy technology. The novel biaxially rotational dynamic radiation therapy (BROAD‐RT) technique of the OXRAY introduces a highly versatile non‐coplanar irradiation modality. BROAD‐RT uses dynamic non‐coplanar beam trajectories via a rotating ring structure, allowing continuous modulation of beam angles during irradiation without requiring patient couch movement.^15^ Preliminary dosimetric analyses suggest that BROAD‐RT substantially reduces the dose to critical OARs, such as the parotid glands and oral cavity, achieving superior CI and HI compared with conventional techniques.^16^ The unique capability of BROAD‐RT to optimize dose distribution across complex anatomical regions makes it particularly suitable for head and neck applications, potentially overcoming the constraints observed in traditional robust planning approaches.

Building on these advancements, we hypothesized that the dynamic non‐coplanar capabilities of BROAD‐RT will enable the creation of dosimetrically superior plans that are robust against positional uncertainties. Therefore, in this study, we aimed to establish the potential benefits of combining robust planning with BROAD‐RT to improve the clinical outcomes of HNC RT by evaluating key dosimetric parameters. To the best of our knowledge, this is the first study to combine BROAD‐RT with robust planning.

## 2 METHODS

### 2.1 Patients and imaging datasets

This retrospective study included 10 patients with oropharyngeal and hypopharyngeal cancers treated with VMAT at the National Cancer Center Hospital East (Table 1). All patients were immobilized using a five‐point thermoplastic mask covering the shoulder and a patient‐specific pillow. The simulation CT datasets were acquired using an Aquilion One scanner (Canon Medical Systems, Tochigi, Japan) with a slice thickness of 2 mm. The requirement for informed consent was waived due to the retrospective nature of the study. Study details were published on the center's homepage, allowing patients to refuse participation. This study, including the investigation procedure and handling of patient information, was approved by the Institutional Review Board IRB No. 2018–076.

**TABLE 1:** Patient characteristics.

Patient	Age, years	Sex	TNM*	CTV high risk Volume (cm^3^)	Site
1	65	Male	T2N0M0	31.04	Hypopharynx
2	58	Male	T4N1M0	63.99	Oropharynx
3	67	Female	T2N1M0	50.66	Oropharynx
4	68	Female	T4N1M0	89.51	Oropharynx
5	55	Female	T2N1M0	44.66	Oropharynx
6	77	Male	T4N2M1	94.72	Oropharynx
7	80	Male	T3N2M0	88.4	Hypopharynx
8	78	Male	T2N1M0	25.16	Oropharynx
9	77	Male	T4N2M0	155.79	Oropharynx
10	70	Male	T4N2M0	113.46	Oropharynx

Abbreviation: CTV, clinical target volume.

^*^8th edition of UICC (Ref. 21).

### 2.2 Contouring

Targets and OARs were delineated by radiation oncologists with expertise in head and neck radiation therapy. Gross tumor volumes (GTVs) of the primary tumors and any lymph node metastases were localized, and a 10 mm margin was added to generate high‐risk clinical target volumes (high‐risk CTVs). Conversely, low‐risk clinical target volumes (low‐risk CTVs) encompassing nodal regions were contoured without additional margin expansion. The OARs included the spinal cord, brainstem, oral cavity, parotid glands, mandible, eyes, cochlea, brain, submandibular glands, and brachial plexus.

### 2.3 Mechanical configuration

The OXRAY features a multileaf collimator (MLC) with a drive speed of 6.50 cm/s, supporting rapid and precise modulation during treatment delivery. The MLC has a leaf thickness of 110 mm, designed to provide high shielding efficiency. The measured MLC transmission rate at our institution was 0.00237. The MLC is composed of central leaves with a width of 2.5 mm and peripheral leaves with a width of 5.0 mm, enabling high‐resolution modulation in the central treatment area. The nominal maximum field size is 20 × 20 cm^2^; however, the gimbal mechanism incorporated into the gantry head allows coverage of a treatment area of up to approximately 30 × 30 cm^2^. The greatest distinguishing feature of the OXRAY system is its clinically available Dynamic Swing Arc (DSA) technique, which enables the O‐ring frame to swing dynamically during irradiation without requiring any movement of the patient couch. The O‐ring can rotate up to ±60°, with user‐defined settings for the swing angle, rotation direction, and modulation control points. In this study, dynamic non‐coplanar trajectories were created within a rotation range from 20° to 340°, allowing continuous irradiation over two full arcs while ensuring collision avoidance with the patient couch. The trajectory path was manually determined by the planner through the specification of gantry and ring angles at each control point. In contrast, the collimator structure in the OXRAY system does not have a rotation mechanism; thus, the collimator angle is fixed at 0° throughout the treatment. In addition, the OXRAY system is equipped with a dual‐kV cone‐beam CT (CBCT) imaging system, featuring two orthogonally arranged x‐ray tubes. This configuration allows for simultaneous acquisition of orthogonal kV images and enables rapid CBCT scan.^16^

### 2.4 Treatment planning

Robust‐VMAT plans were created for comparison with Robust‐BROAD‐RT plans. The target regions were administered two dose levels in 35 fractions with simultaneous integrated boosting (SIB). The prescribed doses were 70 and 56 Gy for high‐ and low‐risk CTVs, respectively. All Robust‐VMAT plans were generated by dosimetrists using the RayStation 2023B Version 14.0 treatment planning system (RaySearch Laboratories AB, Sweden) with a collapsed cone convolution algorithm. The generated plans were subsequently approved by radiation oncologists specialized in HNC and were applied for patient treatment in clinical practice at our institution. Robust‐VMAT plans were created using two full arcs and collimator angles of 350° and 10°. We adopted 6 MV photon beams in this study; non‐coplanar beams were not used in the Robust‐VMAT plans. Dummy structures were placed on both shoulders to avoid incident beams from the shoulder during the optimization calculation. Dose calculation was performed using a grid size of 2 mm.

**FIGURE 1:**
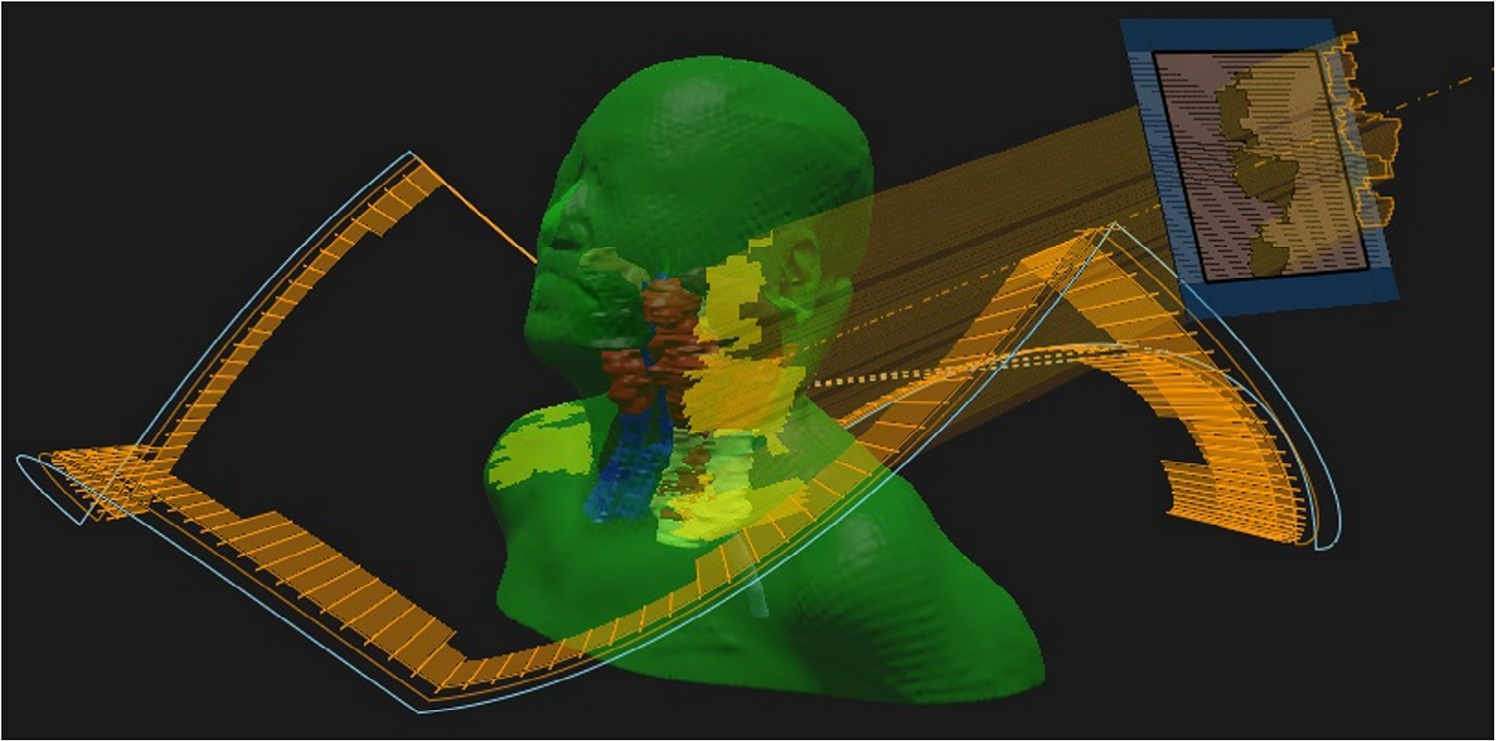
Change in ring angle synchronized with the gantry angle in BROAD‐RT and its arc trajectory. BROAD‐RT, biaxially rotational dynamic radiation therapy.

Additionally, Robust‐Non‐Coplanar‐VMAT (Robust‐NC‐VMAT) plans were created using two full arcs with collimator angles of 10° and 350°, identical to the Robust‐VMAT plans, using a beam energy of 6 MV. The couch was rotated to 20° for the first arc and 340° for the second arc. Although full‐arc irradiation with a 20° couch rotation using a C‐arm linear accelerator poses a high risk of collision with the patient couch and is generally infeasible in clinical practice, we adopted ±20° couch rotations in this study to maintain consistency with non‐coplanar beam geometry used in the BROAD‐RT technique with dynamic trajectory. Robust‐BROAD‐RT plans were created using 6 MV photon beams and two full arcs in the RayStation. The ring structure dynamically rotated between 20° and 340°, generating a continuous non‐coplanar trajectory within this angular range while avoiding collision with the couch or patient. Modulation was performed in six sections per arc to avoid the shoulder (Figure 1). Table S1 summarizes the detailed information of the dynamic trajectories used for BROAD‐RT in this study.

### 2.5 Robustness optimization

Optimization calculations were performed to enhance the robustness of the dose distribution to the clinical target volumes (CTVs) against patient‐setup uncertainties. Patient setup uncertainties of 3–5 mm in each axial direction were considered and incorporated into the optimization. The clinical goals used in the optimization of treatment planning are listed in Table 2. CTV coverage was set at D98 > 97% and D2 < 110%. After plan generation, a robustness evaluation assuming 3 mm setup shifts in each of the six cardinal directions (LR, AP, SI) was performed to ensure consistency in the plan quality of Robust‐VMAT. The results confirmed that the D98 for the CTV did not fall below 95%. Plans that failed to meet the CTV coverage criteria in the worst‐case scenario required replanning. No replanning was performed after the robustness evaluation for the Robust‐NC‐VMAT and Robust‐BROAD‐RT plans to avoid intentional control of robustness outcomes.

**TABLE 2:** Clinical goals for treatment planning.

CTV high risk	D98%	>97%
	D2%	<110%
CTV low risk	D98%	>97%
Spinal cord	Maximum dose	<45 Gy
Brainstem	Maximum dose	<54 Gy
Oral cavity	Mean dose	<30 Gy
Parotid gland_L, Parotid gland_R	Mean dose	<26 Gy (at least one side)
Cochlea_L, Cochlea_R	Mean dose	<40 Gy
Larynx	Mean dose	<45 Gy

*Note*: All dose‐based objectives are expressed as a percentage of the prescription dose.

Abbreviations: CTV, clinical target volume; DX%, dose received by X% of the structure volume.

Plans were created with the highest priority of achieving dose constraints for the spinal cord, brainstem, and CTV. When the clinical goals were met, the dose to OARs was minimized as much as possible. Nominal plans for Robust‐VMAT, Robust‐NC‐VMAT, and Robust‐BROAD‐RT were generated.

### 2.6 Robustness evaluation

Eight uncertainty scenarios were created to evaluate the robustness of all treatment plans generated in this study. These scenarios involved simultaneously applying ±1.7 mm translational shifts along each of the three orthogonal axes—left–right, anterior–posterior, and superior–inferior. This combination yielded a resultant displacement vector of approximately 3 mm. The resulting eight displacement vectors extended from the isocenter to the eight vertices of a virtual cube with a 3 mm length, centered at the isocenter, and each vertex position corresponded to one of the eight robustness evaluation scenarios. This configuration simulated a 3 mm setup error in three‐dimensional space. Each shift scenario was applied to the respective treatment plan, and a worst‐case scenario analysis was conducted. The worst‐case scenario was defined as the one among the eight scenarios in which the dose metric associated with the structure of interest showed the greatest deterioration compared to the nominal plan. This approach is provided as a standard robustness analysis tool in the RayStation.

### 2.7 Dosimetric evaluation

The following dose indices were selected for OAR evaluation in the dosimetric comparison: mean dose to the parotid glands, maximum dose to the spinal cord, mean dose to the oral cavity, dose received by a volume of 1 cc of the mandible (D1cc), maximum dose to the body, and percentage of body volume receiving 105% or more (V105) of the prescribed dose. The selected indices for target evaluation included D98, D50, and D2 of the high‐risk CTV and the CI, HI, and D98 of the low‐risk CTV. CI and HI were calculated as described in Equations (1) and (2), respectively.

(1) $$\text{CI}={\left(\text{TV}\;\_\text{PIV}\right)}^{2}/\left(\mathrm{TV}\times \text{PIV}\right)$$

TV_PIV represents the target volume encompassed by the prescribed isodose volume (PIV), where TV denotes the target volume and PIV represents the volume enveloped by the prescribed isodose.

(2) $$\mathrm{HI}=\left(D2-D98\right)/\mathrm{prescribed}\mathrm{dose}\times 100$$

The dose indices between the Robust‐VMAT, Robust‐NC‐VMAT, and Robust‐BROAD‐RT plans were compared.

### 2.8 Statistical analysis

Dose indices were evaluated using descriptive statistics such as mean and standard deviation. Wilcoxon signed‐rank tests were conducted for comparisons between groups. Statistical significance was set at *p* < 0.05. All statistical analyses were performed using SAS (version 9.4; Cary, NC, USA).

## 3 RESULTS

### 3.1 Dosimetry comparison of Robust‐VMAT and Robust‐BROAD‐RT

**FIGURE 2:**
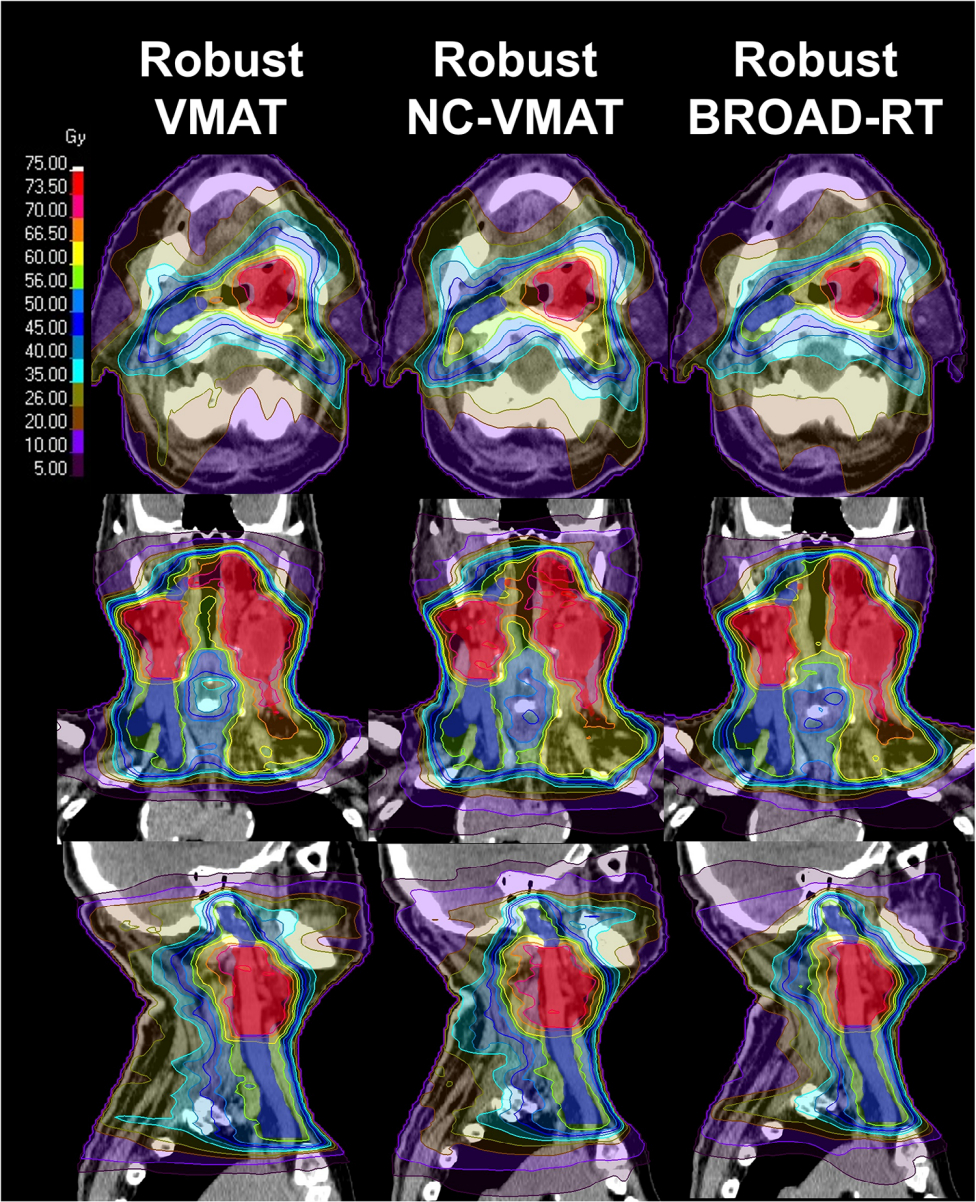
Dose distributions for a representative case in Robust‐VMAT, Robust‐non‐coplanar (NC)‐VMAT, and Robust‐BROAD‐RT. The red region represents the CTV high‐risk structure, and the blue region represents the CTV low‐risk structure. CTV, clinical target volume; VMAT, volumetric modulated arc therapy.

Figure 2 shows the dose distributions for Robust‐VMAT, Robust‐NC‐VMAT, and Robust‐BROAD‐RT plans for a representative case. Table 3 presents a comparison of the dosimetric parameters of the Robust‐VMAT, Robust‐NC‐VMAT, and Robust‐BROAD‐RT plans. No significant difference was observed in D98, D50, D2, CI, and HI between Robust‐VMAT and Robust‐BROAD‐RT plans in the dose coverage index of high‐risk CTV (*p* = 0.084, *p* = 0.11, *p* = 0.77, *p* = 0.477, and *p* = 0.922, respectively). Similarly, no significant differences were observed in D98, D50, CI, or HI between Robust‐BROAD‐RT and Robust‐NC‐VMAT plans (*p* = 0.084, *p* = 0.275, *p* = 0.193, and *p* = 0.812), although a slight improvement was observed in D2 (*p* = 0.038). D98 for the low‐risk CTV was significantly higher with Robust‐BROAD‐RT compared to both Robust‐VMAT and Robust‐NC‐VMAT (*p* = 0.002 for both), potentially reflecting the superior dose‐shaping capability of Robust‐BROAD‐RT. However, the difference is likely of limited clinical impact, as all plans achieved the clinical goal. Among the dosimetric parameters for OARs, the Robust‐BROAD‐RT plans significantly reduced the mean parotid gland dose by 8.17 Gy on the contralateral side and 6.20 Gy on the ipsilateral side (*p* = 0.002, each) and the mean oral cavity dose by 3.03 Gy compared with the Robust‐VMAT plans (*p =* 0.002). A similar significant dose reduction effect on the bilateral parotid glands was observed with non‐coplanar VMAT (*p* = 0.002 and *p* = 0.004, respectively). Although a trend toward dose reduction in the oral cavity was observed, the difference was not statistically significant (*p* = 0.275). Furthermore, the Robust‐BROAD‐RT plans achieved a slight but significant dose reduction compared with the Robust‐NC‐VMAT plans in the contralateral parotid gland (15.37 ± 4.29 Gy vs. 17.93 ± 4.28 Gy, *p* = 0.002), the ipsilateral parotid gland (22.05 ± 6.98 Gy vs. 24.02 ± 7.74 Gy, *p* = 0.002), and the oral cavity (25.52 ± 9.45 Gy vs. 27.71 ± 10.25 Gy, *p* = 0.02). No significant differences were observed in the maximum dose indices of the brainstem and mandible between the Robust‐VMAT, Robust‐NC‐VMAT, and Robust‐BROAD‐RT plans. The Robust‐BROAD‐RT plans significantly reduced the maximum dose to the spinal cord compared with both the Robust‐VMAT (43.03 ± 1.29 Gy vs. 41.47 ± 1.12 Gy, *p* = 0.002) and the Robust‐NC‐VMAT plans (42.86 ± 1.55 Gy vs. 41.47 ± 1.12 Gy, *p* = 0.02). No significant differences were observed in the overdose region between the Robust‐BROAD‐RT and Robust‐VMAT plans in terms of the maximum doses to the body and V105. However, a slight increase in V105 was observed with the Robust‐NC‐VMAT plans compared to both the Robust‐VMAT and BROAD‐RT plans (*p* = 0.004 and *p* = 0.02, respectively). The Robust‐NC‐VMAT and Robust‐BROAD‐RT plans had significantly longer delivery times (150.7 and 157.4 s vs. 127.8 s, *p* = 0.004 and *p* = 0.011) than the Robust‐VMAT planes. The Robust‐BROAD‐RT plans exhibited a trend toward higher total monitor unit (MU) values compared to the Robust‐VMAT plans (710.95 ± 66.63 vs. 651.59 ± 106.01), although this difference did not reach statistical significance (*p* = 0.131). In contrast, a significant increase in total MU was observed when compared to the Robust‐NC‐VMAT plans (*p* = 0.004).

**TABLE 3:** Comparison dose indices, delivery time, and total MU between Robust‐VMAT, Robust‐NC‐VMAT, and Robust‐BROAD‐RT plans.

		Robust‐VMAT (mean ± SD)	Robust‐NC‐VMAT (mean ± SD)	Robust‐BROAD‐RT (mean ± SD)	*p*‐values (NC‐VMAT vs. VMAT)	*p*‐values (BROAD‐RT vs. VMAT)	*p*‐values (BROAD‐RT vs. NC‐VMAT)
CTV high risk							
	D98 (%)	99.9 ± 0.59	99.9 ± 0.81	100.35 ± 0.97	0.77	0.084	0.084
	D50 (%)	102.45 ± 0.33	102.54 ± 0.43	102.63 ± 0.30	0.492	0.11	0.275
	D2 (%)	104.32 ± 0.31	104.79 ± 0.34	104.30 ± 0.37	0.006	0.77	0.038
	CI	0.65 ± 0.07	0.62 ± 0.09	0.67 ± 0.13	0.275	0.477	0.193
	HI	0.84 ± 0.09	0.85 ± 0.07	0.84 ± 0.11	0.888	0.922	0.812
CTV low risk							
	D98 (%)	99.53 ± 0.46	99.9 ± 1.15	100.90 ± 0.90	0.232	0.002	0.002
Contralateral parotid gland							
	Mean dose (Gy)	23.54 ± 2.53	17.93 ± 4.28	15.37 ± 4.29	0.002	0.002	0.002
Ipsilateral parotid gland							
	Mean dose (Gy)	28.25 ± 7.71	24.02 ± 7.74	22.05 ± 6.98	0.004	0.002	0.006
Contralateral submandibular gland							
	Mean dose (Gy)	50.30 ± 7.74	46.22 ± 10.96	47.97 ± 9.32	0.064	0.084	0.131
Ipsilateral submandibular gland							
	Mean dose (Gy)	64.30 ± 8.04	64.02 ± 8.71	63.32 ± 7.72	1	0.432	0.432
Oral cavity							
	Mean dose (Gy)	28.55 ± 9.76	27.71 ± 10.25	25.52 ± 9.45	0.275	0.006	0.02
Spinal cord							
	D1cc (Gy)	37.93 ± 2.09	37.97 ± 1.58	37.59 ± 1.81	0.77	0.846	0.492
	Dmax (Gy)	43.03 ± 1.29	42.86 ± 1.55	41.47 ± 1.12	0.77	0.002	0.02
Brainstem							
	Dmax (Gy)	38.63 ± 13.90	39.95 ± 10.02	37.14 ± 11.80	0.492	0.77	0.139
Mandible							
	D1cc (Gy)	58.77 ± 9.90	61.22 ± 11.41	59.10 ± 9.94	0.049	0.77	0.105
Larynx							
	Mean dose (Gy)	35.45 ± 12.24	40.25 ± 11.58	38.73 ± 11.30	0.011	0.014	0.131
Brachial plexus L							
	Dmax (Gy)	58.02 ± 6.07	56.44 ± 6.41	56.61 ± 6.44	0.131	0.232	0.695
Brachial plexus R							
	Dmax (Gy)	57.04 ± 3.20	55.29 ± 6.83	54.07 ± 9.12	0.322	0.105	0.232
Body							
	Dmax (%)	105.68 ± 0.56	106.51 ± 0.72	105.85 ± 0.44	0.014	0.77	0.064
	V105 (cm^3^)	0.32 ± 0.49	1.14 ± 0.58	0.45 ± 0.43	0.004	0.625	0.02
Delivery time (s)		127.80 ± 9.37	150.70 ± 0.82	157.40 ± 15.85	0.004	0.011	0.131
Total MU		651.59 ± 106.01	484.27 ± 61.22	710.95 ± 66.63	0.002	0.131	0.004

Abbreviations: BROAD‐RT, biaxially rotational dynamic radiation therapy; CI, Conformity Index; CTV, clinical target volume; Dmax, maximum dose; DX, dose received by the X% of the volume; Gy, gray; HI, Homogeneity Index; MU, monitor unit; NC, non‐coplanar; Robust‐NC‐VMAT, Robust‐Non‐Coplanar‐VMAT; VMAT, volumetric modulated arc therapy; VX, the organ volume that received by X% of the prescription dose or more.

### 3.2 Robustness in the worst‐scenario

**FIGURE 3:**
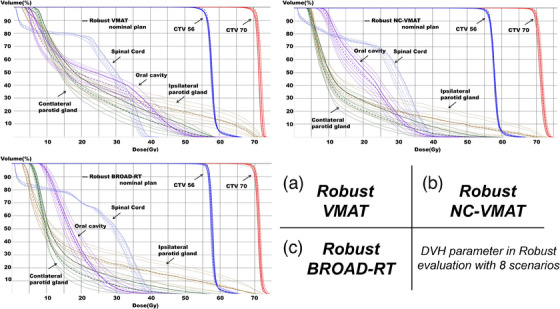
DVHs under robust evaluation with eight setup uncertainty scenarios for three different planning techniques: (a) Robust‐VMAT, (b) Robust‐NC‐VMAT, and (c) Robust‐BROAD‐RT. Nominal plans are shown as dashed lines. Solid lines represent DVH distributions under worst‐case perturbations in target and OAR positions. Target volumes (CTV56 and CTV70) are indicated in red and blue, respectively. Key OARs include the spinal cord, oral cavity, and bilateral parotid glands. CTV, clinical target volume; DVH, dose–volume histogram; OAR, organ at risk; VMAT, volumetric modulated arc therapy.

Figure 3 presents dose–volume histograms (DVHs) comparing the eight scenarios for the representative case. Table 4 presents the comparison of the Robust‐VMAT, Robust‐NC‐VMAT, and Robust‐BROAD‐RT plans in terms of dosimetric parameters in the worst‐case scenario. No significant difference was observed in D98, D50, D2, CI, or HI between the Robust‐VMAT, Robust‐NC‐VMAT, and Robust‐BROAD‐RT plans in terms of dose coverage metrics for the high‐risk CTV. Similarly, no significant difference was observed in D98 between the Robust‐VMAT, Robust‐NC‐VMAT, and Robust‐BROAD‐RT for the low‐risk CTV (*p* = 0.432, *p* = 0.77, and *p* = 0.432). Among the dosimetric parameters for OARs, the Robust‐BROAD‐RT plans significantly reduced the mean parotid gland dose by 8.11 Gy on the contralateral side and 6.25 Gy on the ipsilateral side (*p* = 0.002, each) and the mean oral cavity dose by 3.18 Gy compared with the Robust‐VMAT plans (*p* = 0.002). Similarly, the dose‐sparing effect for both parotid glands was maintained in the Robust‐NC‐VMAT plans compared with the Robust‐VMAT plans (*p* = 0.002, each). However, no significant difference was observed for the oral cavity dose (*p* = 0.232). The dose‐sparing effect for both parotid glands and the oral cavity was maintained in the Robust‐BROAD‐RT plans compared with the Robust‐NC‐VMAT plans, with lower mean doses observed for the contralateral parotid gland (19.20 ± 4.76 Gy vs. 21.80 ± 5.03 Gy, *p* = 0.004), the ipsilateral parotid gland (26.39 ± 7.63 Gy vs. 28.16 ± 8.34 Gy, *p* = 0.014), and the oral cavity (28.10 ± 10.41 Gy vs. 30.34 ± 10.96 Gy, *p* = 0.014). No significant differences were observed in the maximum dose indices for the spinal cord and mandible between the Robust‐VMAT, Robust‐NC‐VMAT, and Robust‐BROAD‐RT plans. In contrast, the maximum dose to the brainstem was significantly lower in the Robust‐BROAD‐RT plans compared with the Robust‐NC‐VMAT plans (*p* = 0.02). No significant differences were observed in the overdose region between the Robust‐VMAT, Robust‐NC‐VMAT, and Robust‐BROAD‐RT plans in terms of the maximum doses to the body and V105.

**TABLE 4:** Comparison dose indices between Robust‐VMAT, Robust‐NC‐VMAT, and Robust‐BROAD‐RT plans in the worst case scenario.

		Robust‐VMAT (Mean ± SD)	Robust‐NC‐VMAT (Mean ± SD)	Robust‐BROAD‐RT (Mean ± SD)	*p*‐values (NC‐VMAT vs. VMAT)	*p*‐values (BROAD‐RT vs. VMAT)	*p*‐values (BROAD‐RT vs. NC‐VMAT)
CTV high risk							
	D98 (%)	97.83 ± 1.32	98.29 ± 1.34	97.84 ± 2.15	0.492	0.846	0.695
	D50 (%)	101.92 ± 0.30	102.09 ± 0.53	102.05 ± 0.35	0.432	0.375	0.846
	D2 (%)	104.91 ± 0.32	104.69 ± 0.98	104.84 ± 0.49	0.695	0.77	1
	CI	0.61 ± 0.07	0.59 ± 0.08	0.63 ± 0.11	0.275	0.4	0.193
	HI	0.70 ± 0.12	0.71 ± 0.10	0.67 ± 0.14	0.922	0.7	0.205
CTV low risk							
	D98 (%)	97.21 ± 0.63	97.26 ± 2.29	97.40 ± 2.81	0.432	0.77	0.432
Contralateral parotid gland							
	Mean dose (Gy)	27.31 ± 2.98	21.80 ± 5.03	19.20 ± 4.76	0.002	0.002	0.004
Ipsilateral parotid gland							
	Mean dose (Gy)	32.64 ± 8.34	28.16 ± 8.34	26.39 ± 7.63	0.002	0.002	0.014
Contralateral submandibular gland							
	Mean dose (Gy)	52.55 ± 7.60	52.21 ± 11.14	51.14 ± 9.20	0.625	0.275	0.557
Ipsilateral submandibular gland							
	Mean dose (Gy)	66.01 ± 7.24	62.97 ± 11.04	65.34 ± 7.31	0.232	0.432	0.557
Oral cavity							
	Mean dose (Gy)	31.28 ± 10.38	30.34 ± 10.96	28.10 ± 10.41	0.232	0.002	0.014
Spinal cord							
	D1cc (Gy)	39.41 ± 1.90	39.48 ± 1.86	39.09 ± 1.42	1	0.695	0.625
	Dmax (Gy)	47.03 ± 2.58	46.70 ± 3.20	45.99 ± 1.14	0.695	0.275	0.557
Brainstem							
	Dmax (Gy)	42.85 ± 14.61	44.27 ± 12.55	40.64 ± 14.10	0.322	0.557	0.02
Mandible							
	D1cc (Gy)	62.35 ± 8.96	64.04 ± 10.45	62.44 ± 8.98	0.064	0.922	0.16
Larynx							
	Mean dose (Gy)	37.89 ± 12.45	42.24 ± 11.87	40.65 ± 11.69	0.01	0.027	0.084
Brachial plexus L							
	Dmax (Gy)	59.64 ± 5.16	58.37 ± 5.09	58.75 ± 4.81	0.131	0.232	0.193
Brachial plexus R							
	Dmax (Gy)	58.20 ± 2.78	56.43 ± 6.53	55.83 ± 7.46	0.322	0.232	0.432
Body							
	Dmax (%)	106.98 ± 0.87	107.00 ± 0.86	106.87 ± 0.64	0.922	0.77	0.93
	V105 (cm^3^)	1.90 ± 1.37	2.63 ± 1.59	1.89 ± 1.44	0.275	1	0.275

Abbreviations: BROAD‐RT, biaxially rotational dynamic radiation therapy; CI, Conformity Index; CTV, clinical target volume; Dmax, maximum dose; DX, dose received by the X% of the volume; Gy, gray; HI, Homogeneity Index; MU, monitor unit; NC, non‐coplanar; Robust‐NC‐VMAT, Robust‐Non‐Coplanar‐VMAT; VMAT, volumetric modulated arc therapy; VX, the organ volume that received by X% of the prescription dose or more.

**FIGURE 4:**
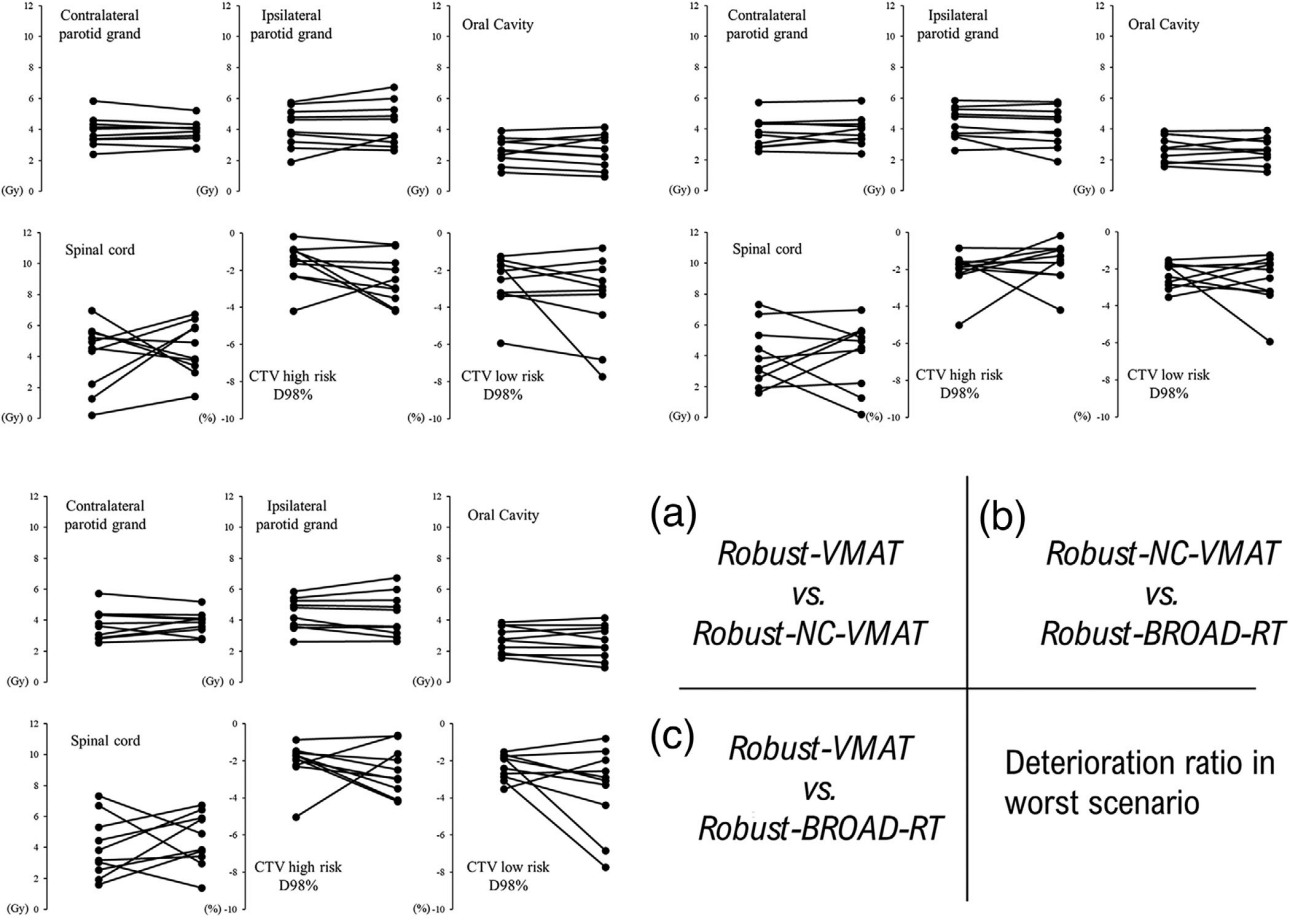
Absolute differences in deterioration values under worst‐case scenarios for OAR doses and CTV D98 values under setup uncertainties across all patients. Each line represents an individual patient, with deterioration values compared between paired planning techniques: (a) Robust‐VMAT (left) versus Robust‐NC‐VMAT (right), (b) Robust‐NC‐VMAT (left) versus Robust‐BROAD‐RT (right), (c) Robust‐VMAT (left) versus Robust‐BROAD‐RT (right). Deterioration is defined as the increase in OAR dose (Gy) or decrease in CTV D98 (%) in the worst‐case scenario relative to the nominal plan. BROAD‐RT, biaxially rotational dynamic radiation therapy; CTV, clinical target volume; OAR, organ at risk; VMAT, volumetric modulated arc therapy.

Figure 4 shows the deterioration ratio between the nominal and worst‐case plans for Robust‐VMAT, Robust‐NC‐VMAT, and Robust‐BROAD‐RT. A slight deterioration was observed in the D98 dose coverage for the low‐risk CTV with Robust‐NC‐VMAT and Robust‐ BROAD‐RT; however, the deterioration rates for all dose parameters did not differ significantly.

## 4 DISCUSSION

In this study, we aimed to establish the potential benefits of combining robust planning with BROAD‐RT and found that the Robust‐BROAD‐RT plans significantly reduced the dose to the parotid glands and oral cavity compared with the Robust‐VMAT plans, while maintaining the CTV coverage and CI/HI. However, the Robust‐BROAD‐RT plans exhibited an increase in treatment time compared with the Robust‐VMAT plans. This finding is consistent with previously reported planning studies using the OXRAY system for oropharyngeal and hypopharyngeal cancers.^16^

The increase in treatment time was attributed to the rotation of the ring structure, which is a characteristic feature of BROAD‐RT. The dynamic trajectory used in the study included a maximum ring rotation of ±20°. In conventional non‐coplanar VMAT using a C‐arm linear accelerator, it is necessary to rotate the patient couch by approximately 20° and 340° to achieve beam angles equivalent to those generated by BROAD‐RT. In clinical practice, rotating the couch by 20° during full‐arc VMAT delivery frequently results in collision between the gantry and the couch, making it impractical to generate a complete full‐arc plan under these conditions. Consequently, the use of a specialized extended couch would be required to implement such non‐coplanar VMAT trajectories clinically. In contrast, the dynamic trajectories enabled by the OXRAY system provide high versatility and avoid collision risks by ensuring a sufficient clearance between the couch and the gantry during dynamic beam delivery. Even if a specialized couch was employed, full‐arc non‐coplanar VMAT with a ±20° couch rotation would still encounter a significantly reduced clearance, particularly when the gantry approaches ±90°, where the risk of collision is maximal. Therefore, rotating the couch by approximately ±20° during beam delivery is important to achieve optimal beam trajectories because lateral beam entry is critical for optimizing parotid gland sparing. However, due to mechanical limitations, the feasibility of implementing such couch rotations in clinical practice is substantially restricted. Thus, the dynamic swing approach utilized by BROAD‐RT offers a clinically advantageous alternative by enabling collision‐free, non‐coplanar full‐arc delivery without requiring patient couch rotation.

The treatment time for Robust‐BROAD‐RT was approximately 30 s longer than that for Robust‐VMAT, which is a minor cost for significant dose reduction to the parotid glands and oral cavity, since the benefits of improved dose parameters outweigh this increase. It should be noted that BROAD‐RT and NC‐VMAT carry the risk of increased doses of OARs outside the treatment region because of the use of non‐coplanar beams. Table S2 presents the dose parameters for the eyes, brain, cochlea, and low‐dose exposure volumes in the Robust‐VMAT, Robust‐NC‐VMAT, and Robust‐BROAD‐RT plans. The mean and maximum doses to the eyes as well as the low‐dose volume parameters V10, V5, and V1 were significantly increased in Robust‐NC‐VMAT and Robust‐BROAD‐RT. Moreover, the difference from coplanar VMAT became more pronounced when the dose decreased to less than 10 Gy. This finding is consistent with the observations of Gayen et al. in their study on non‐coplanar VMAT for HNC.^12^

In addition, the dose distribution stability was evaluated using the worst‐case scenario method to compare robustness. A few cases showed deteriorated D98 dose coverage in Robust‐NC‐VMAT and Robust‐BROAD‐RT compared with conventional Robust‐VMAT; however, no statistically significant differences were found. Additionally, no significant differences were observed in D50 or CI/HI regarding the deterioration of target‐related metrics. Although the Robust‐VMAT and Robust‐BROAD‐RT plans showed deterioration in dose metrics under the worst‐case scenario, no statistically significant differences in the degree of deterioration were observed for the OARs (Table S3). This finding is potentially related to previous studies on the dynamic trajectory radiation therapy (DTRT), another dynamic trajectory irradiation method similar to BROAD‐RT in OXRAY.^17^, ^18^

**FIGURE 5:**
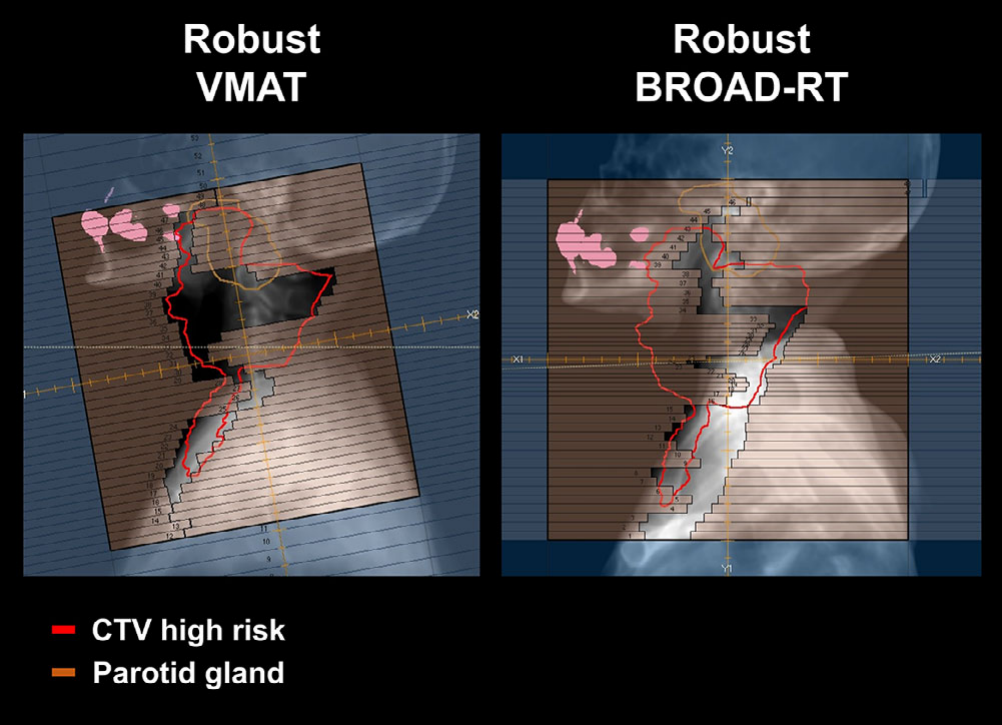
Avoiding overlap of the parotid gland and the target by dynamic trajectory of biaxially rotational dynamic radiation therapy.

Loebner et al. evaluated the robustness of DTRT and reported that the D98 coverage was more susceptible to patient setup uncertainties and MLC positional errors than VMAT.^18^ Similarly, in the present study, we observed a tendency for a lower D98 coverage in the Robust‐BROAD‐RT plan than in the conventional Robust‐VMAT plan in some cases in the robustness evaluation. In particular, a deterioration of more than 5% in the worst‐case scenario compared with the nominal plan was observed in two cases for the D98 of the low‐risk CTV. In both cases, the low‐risk CTV was adjacent to and covered the parotid glands. Although reducing the dose to the parotid gland is challenging in the nominal plan of Robust‐VMAT, with Robust‐BROAD‐RT, a steep dose gradient could be created at the boundary between the parotid glands and CTV, significantly reducing the mean dose to the parotid glands. Nevertheless, this steep dose gradient became a drawback in the worst‐case scenario (when the patient shifted cranially), leading to a deterioration in the CTV coverage around the parotid glands. This trend suggests that while dynamic trajectory irradiation methods, such as DTRT and BROAD‐RT, offer improved OAR sparing and dose conformity, they may be more sensitive to certain types of geometric uncertainties than conventional coplanar techniques. These findings are also consistent with those of Loebner et al., who found that the dose to the parotid glands and oral cavity was significantly reduced in DTRT compared with VMAT. The superior sparing of the OARs in DTRT and Robust‐BROAD‐RT is likely due to the greater flexibility of the beam trajectory, which allows better avoidance of critical structures (Figure 5). Therefore, even under the worst‐case scenario, the dose‐reduction effect on the parotid glands and oral cavity was maintained in the Robust‐BROAD‐RT plan, with significantly lower doses observed compared with the Robust‐VMAT plan.

Bertholet et al. demonstrated that robust optimization can improve the stability of dynamic non‐coplanar techniques such as DTRT.^19^ Although BROAD‐RT employs a different delivery mechanism, these findings support the application of robust planning to dynamic trajectory‐based irradiation. To our knowledge, this study is the first to implement robust optimization specifically for BROAD‐RT on the OXRAY platform. The proposed combination of robust planning with BROAD‐RT demonstrated the potential to achieve further dose reduction to the surrounding OARs while maintaining a robustness comparable to that of conventional Robust‐VMAT. However, current robust planning algorithms may not accurately account for the robustness of dynamic trajectory irradiation, which could explain the observed reduction in D98 coverage compared with the conventional Robust‐VMAT approach. Therefore, future advancements in robust planning algorithms tailored to dynamic trajectory irradiation are required.

Moreover, no significant differences were observed between the Robust‐BROAD‐RT and Robust‐VMAT plans for nearly all OAR dose parameters. This may be due to the absence of robust optimization for the spinal cord in the treatment planning process employed in this study. In robust optimization, the spinal cord is often included as a robust structure alongside the CTV due to its clinical importance. Although the present study primarily focused on investigating the relationship between robust optimization for the CTV and dynamic trajectory irradiation, we conducted an additional planning analysis in which the spinal cord was included in the robust optimization process. Table S4 summarizes a comparative analysis of the degradation in key dose metrics between the nominal and worst‐case scenarios for Robust‐BROAD‐RT and Robust‐BROAD‐RT with Robust OAR optimization (specifically, the spinal cord). The robust optimization parameters and priority level for the spinal cord were set to be equivalent to those for the CTV. The results showed that incorporating robust optimization for the spinal cord resulted in a slight reduction in the degree of dose degradation for the spinal cord under setup uncertainty; however, this difference did not reach statistical significance. Furthermore, trade‐offs were observed, including a slight increase in oral cavity dose and reductions in target coverage metrics in some cases. Robust optimization aims to maintain the stability of specific dose metrics under geometric uncertainty by explicitly optimizing the associated structure. However, as seen in the relationship between the low‐risk CTV and the parotid glands, when two adjacent structures are simultaneously subjected to strong optimization constraints, the intended robustness may not be fully realized. Thus, robust optimization should be regarded as one of the several tools available for shaping dose distributions. Moreover, the achieved robustness is influenced by other planning techniques and the relative strength of dose constraints applied to each organ. Therefore, it is important to recognize that robustness is not guaranteed simply by applying robust optimization with the highest priority. Robust‐BROAD‐RT demonstrated a high capability for dose shaping and was particularly effective in reducing the dose to the parotid glands adjacent to the CTV. However, in clinical application, attention must be paid to the risk of recurrence from targets near the parotid region. Sari et al. analyzed the risk and characteristics of parotid region recurrence in 746 patients with nasopharyngeal carcinoma over a 30‐year period.^20^ Their analysis identified five cases of parotid or periparotid recurrence, four of which occurred in patients treated with parotid‐sparing IMRT. Similarly, in this study, the presence of level II lymph node metastasis exceeding 2 cm and retropharyngeal lymph node metastasis were identified as significant risk factors. Therefore, when performing parotid‐sparing IMRT using Robust‐BROAD‐RT, a prior robustness evaluation should be conducted to confirm the stability of adjacent targets under uncertainty before clinical implementation.

Despite the positive outcomes, this study has some limitations. First, the range of uncertainty applied to the robust plans varied between 3 and 5 mm for each patient and could not be standardized. Second, the number of cases analyzed in this study was limited to 10, posing constraints on statistical interpretation. Third, all virtual plans in this study were created by a single planner, which may have influenced the results due to planning experience and methodological variations. Fourth, robust evaluation was performed using the setup uncertainty simulation function available in RayStation, which models positional deviations of the patient. The RayStation also supports anatomical robustness simulations, which allow for evaluation of dose variations in response to expected anatomical changes—such as parotid gland shrinkage. However, these anatomical scenarios were not incorporated into the robust evaluation in this study; therefore, further investigation is warranted to assess the robustness of dose distributions under such conditions. Nevertheless, the present study reinforces the findings of previous robustness analyses of dynamic trajectory irradiation methods, demonstrating their benefits in OAR sparing while highlighting their potential vulnerabilities in target coverage robustness. Although robust optimization enhances resilience to geometric uncertainties, over‐shaping of dose gradients—such as the creation of excessively steep dose fall‐off between the CTV and adjacent OARs—may compromise plan robustness and should be carefully managed. In particular, the high dose‐shaping capability of Robust‐BROAD‐RT, which is otherwise a major advantage, may inadvertently result in decreased CTV coverage in certain cases due to the formation of steep dose gradients near critical structures like the parotid glands. Therefore, robust optimization does not inherently guarantee adequate CTV coverage, and it is essential to perform robust evaluation to ensure that the plan remains resilient against the intended range of positional uncertainties. To fully exploit the clinical advantages of Robust‐BROAD‐RT—similar to conventional head and neck IMRT—rigorous implementation of IGRT, precise patient positioning, and adaptive radiotherapy (ART) protocols remain essential components of safe and effective treatment delivery. Ultimately, while our study demonstrates the clear benefit of Robust‐BROAD‐RT in reducing OAR doses, a small subset of cases exhibited compromised CTV coverage. This highlights the importance of carefully balancing target coverage and OAR sparing under robust optimization constraints. When implemented with prior robustness evaluation and due consideration of these limitations, Robust‐BROAD‐RT is expected to ensure target coverage while achieving further reduction in OAR doses in head and neck radiotherapy.

## 5 CONCLUSION

Robust‐BROAD‐RT achieved significant dose reductions to the parotid glands and oral cavity compared with Robust‐VMAT. Notably, these dose‐sparing benefits were preserved even under the worst‐case scenario, with CTV coverage degradation remaining comparable to Robust‐VMAT. These findings suggest that the application of robust optimization to BROAD‐RT may offer substantial dosimetric advantages while maintaining robustness comparable to conventional VMAT. However, further validation with larger and more heterogeneous cohorts is needed to confirm these preliminary findings.

## AUTHOR CONTRIBUTIONS

*Concept and design*: Kouta Hirotaki and Kana Motegi. *Treatment planning creation*: Kouta Hirotaki, *Data analysis, and interpretation*: Kouta Hirotaki and Masashi Wakabayashi. *Statistical analysis*: Masashi Wakabayashi. *Important advice and critical discussion*: Kana Motegi, Shunsuke Moriya, and Sadamoto Zenda. *Review of dose distributions (radiation oncologists)*: Sadamoto Zenda, *Research management and supervision*: Masashi Ito and Takeji Sakae. All the authors have read and approved the final version of the manuscript.

## CONFLICT OF INTEREST STATEMENT

Kouta Hirotaki reports receiving speaking and lecture fees from Hitachi High‐Tech Ltd. The other authors declare no conflicts of interest.

## ETHICS APPROVAL

This study, including the investigation procedure and handling of patient information, was approved by the Institutional Review Board of National Cancer Center Hospital East (IRB No. 2018–076).

## Supporting information



Supporting Information

Supporting Information

## Data Availability

The datasets generated and/or analyzed during this study are not publicly available due to institutional and ethical restrictions. Access to the data may be granted upon reasonable request and with approval from the Institutional Review Board.
